# The Impact of ESCRT on Aβ_1-42_ Induced Membrane Lesions in a Yeast Model for Alzheimer’s Disease

**DOI:** 10.3389/fnmol.2018.00406

**Published:** 2018-11-05

**Authors:** Gernot Fruhmann, Christelle Marchal, Hélène Vignaud, Mathias Verduyckt, Nicolas Talarek, Claudio De Virgilio, Joris Winderickx, Christophe Cullin

**Affiliations:** ^1^Functional Biology, KU Leuven, Leuven, Belgium; ^2^Institut de Chimie et Biologie des Membranes et des Nano-objets, University of Bordeaux, CNRS UMR 5248, Pessac, France; ^3^Institut de Génétique Moléculaire de Montpellier, University of Montpellier, CNRS, Montpellier, France; ^4^Department of Biology, Université de Fribourg, Fribourg, Switzerland

**Keywords:** Aβ_42_, amyloid beta, Alzheimer’s disease, ESCRT, membrane repair, *Saccharomyces cerevisiae*, yeast

## Abstract

Aβ metabolism plays a pivotal role in Alzheimer’s disease. Here, we used a yeast model to monitor Aβ_42_ toxicity when entering the secretory pathway and demonstrate that processing in, and exit from the endoplasmic reticulum (ER) is required to unleash the full Aβ_42_ toxic potential. Consistent with previously reported data, our data suggests that Aβ_42_ interacts with mitochondria, thereby enhancing formation of reactive oxygen species and eventually leading to cell demise. We used our model to search for genes that modulate this deleterious effect, either by reducing or enhancing Aβ_42_ toxicity, based on screening of the yeast knockout collection. This revealed a reduced Aβ_42_ toxicity not only in strains hampered in ER-Golgi traffic and mitochondrial functioning but also in strains lacking genes connected to the cell cycle and the DNA replication stress response. On the other hand, increased Aβ_42_ toxicity was observed in strains affected in the actin cytoskeleton organization, endocytosis and the formation of multivesicular bodies, including key factors of the ESCRT machinery. Since the latter was shown to be required for the repair of membrane lesions in mammalian systems, we studied this aspect in more detail in our yeast model. Our data demonstrated that Aβ_42_ heavily disturbed the plasma membrane integrity in a strain lacking the ESCRT-III accessory factor Bro1, a phenotype that came along with a severe growth defect and enhanced loading of lipid droplets. Thus, it appears that also in yeast ESCRT is required for membrane repair, thereby counteracting one of the deleterious effects induced by the expression of Aβ_42_. Combined, our studies once more validated the use of yeast as a model to investigate fundamental mechanisms underlying the etiology of neurodegenerative disorders.

## Introduction

Amyloid β_1-42_ (referred to as Aβ_42_) is an intensively investigated peptide involved in Alzheimer’s disease (AD) but beyond its role as a possibly malignant factor, the physiological functions of Aβ_42_ are still speculative. It has been shown that it has a role in activity dependent synaptic vesicle release and there is evidence that it might act as an antimicrobial agent in the brain, but besides that, not much is known about its physiological function (Pearson and Peers, 2006;Abramov et al., 2009; Kagan et al., 2012; Kumar et al., 2016). Thus, although several efforts have been put in investigating its role in AD, still most of its functions remain elusive. In humans, the membrane bound amyloid precursor protein, APP, is processed in two possible ways: it can enter the non-amyloidogenic pathway or it might be cleaved by β- and γ-secretases in the amyloidogenic pathway thus producing Aβ peptides of lengths between 37 and 43 amino acids (LaFerla et al., 2007). In AD, these peptides are then secreted and form inert extracellular plaques, but monomers and especially small oligomers are transported to several intracellular organelles where they reveal their toxic potential. For instance, strong evidence was found for that Aβ_42_ perturbs proper function of mitochondria through blocking respiration at complex IV, resulting in cells producing more reactive oxygen species (ROS) and eventually apoptotic cell death (Wang et al., 2010; Ittner and Gotz, 2011; Benilova et al., 2012; Jackrel et al., 2014; Alexandrov et al., 2016; Vicente Miranda et al., 2016).

In the past decade, yeast was established as a model organism for studying fundamental aspects related to neurodegenerative disorders such as Huntington’s, Parkinson’s, or AD (Winderickx et al., 2008; Franssens et al., 2010; Swinnen et al., 2011; Porzoor and Macreadie, 2013; Jiang et al., 2016; Verduyckt et al., 2016; Fruhmann et al., 2017). Since most of the overall cellular architecture and most basic biochemical processes are conserved between mammalian cells and yeast, and given the fact that about 20–30 per cent of all human genes have orthologs in *Saccharomyces cerevisiae* (Foury, 1997; Hamza et al., 2015; Liu et al., 2017), it is obvious that certain disease mechanisms can be studied in this easy to handle model organism. In connection to AD and Aβ_42_, we and others reported that expression of this peptide triggers toxicity in yeast when targeted to the secretory pathway as to mimic its multi-compartment trafficking observed in mammalian systems. This led to the observation that Aβ_42_ expression in yeast alters endocytosis of plasma membrane resident proteins (Treusch et al., 2011; D’Angelo et al., 2013), induces ER-stress and the unfolded protein response (Chen et al., 2017) and triggers mitochondrial dysfunction (D’Angelo et al., 2013; Chen and Petranovic, 2015; Chen et al., 2017). In the studies presented in this paper, we used previously reported constructs (D’Angelo et al., 2013; Vignaud et al., 2013) where the yeast mating type α-prepro factor directs Aβ_42_ into the Golgi. Here, the α-prepro factor is cleaved off, followed by transport of the peptide to the plasma membrane. In addition, the constructs contain a C-terminal linker-GFP tag in order to ensure stable expression and easy localization of Aβ_42_ in the yeast cells. Besides the wild-type Aβ_42_ and the clinical arctic mutant, we also expressed two synthetic mutants generated by random mutagenesis and previously shown to be either more toxic (Aβ_42_G37C) to, or to be moderately toxic (Aβ_42_L34T) compared to Aβ_42_wt (D’Angelo et al., 2013; Vignaud et al., 2013). Using these constructs, we performed genome-wide screenings as to identify Aβ_42_ toxicity modulators. We confirm the previously reported Aβ_42_ toxicity phenotypes and in addition demonstrate that Aβ_42_ introduces membrane lesions that require the ESCRT system in order to become repaired.

## Materials and Methods

### Yeast Strains, Plasmids, and Media

We used the haploid *Saccharomyces cerevisiae* strain BY4741 MATa *his3Δ1 leu2Δ0 met15Δ0 ura3Δ0* and BY4742 MATα *his3Δ1 leu2Δ0 lys2Δ0 ura3Δ0* for all specified experiments. All deletion strains were obtained from the commercial EUROSCARF knock-out library (Y.K.O. collection). For a full list of strains used in this study see Table 1.

**Table 1:** Yeast strains used in this study.

Name	Genotype	Source
Query strain (SGA)	MATα *can1Δ::STEpr-HIS5sp lyp1Δ his3Δ1 leu2Δ0 met15Δ0 ura3Δ0 LYS2*	Tong et al., 2001
Deletion mutant strains (SGA)	MATa *Target_gene::kanMX4 his3Δ1 leu2Δ0 met15Δ0 ura3Δ0*	Tong et al., 2001
BY4741	MATa *his3Δ1 leu2Δ0 met15Δ0 ura3Δ0*	Openbiosystems
BY4742	MATα *his3Δ1 leu2Δ0 lys2Δ0 ura3Δ0*	Openbiosystems
JW 12 918	MATa *his3Δ1 leu2Δ0 met15Δ0 ura3Δ0 erv29::kanMX*	Y.K.O. collection
JW 23 168	MATα *his3Δ1 leu2Δ0 lys2Δ0 ura3Δ0 hse1Δ::kanMX*	Y.K.O. collection
JW 23 771	MATα *his3Δ1 leu2Δ0 lys2Δ0 ura3Δ0 vps27::kanMX*	Y.K.O. collection
JW 20 178	MATα *his3Δ1 leu2Δ0 lys2Δ0 ura3Δ0 srn2::kanMX*	Y.K.O. collection
JW 21 184	MATα *his3Δ1 leu2Δ0 lys2Δ0 ura3Δ0 mvb12::kanMX*	Y.K.O. collection
JW 21 335	MATα *his3Δ1 leu2Δ0 lys2Δ0 ura3Δ0 stp22::kanMX*	Y.K.O. collection
JW 22 115	MATα *his3Δ1 leu2Δ0 lys2Δ0 ura3Δ0 vps28::kanMX*	Y.K.O. collection
JW 23 142	MATα *his3Δ1 leu2Δ0 lys2Δ0 ura3Δ0 vps25::kanMX*	Y.K.O. collection
JW 21 849	MATα *his3Δ1 leu2Δ0 lys2Δ0 ura3Δ0 vps36::kanMX*	Y.K.O. collection
JW 22 164	MATα *his3Δ1 leu2Δ0 lys2Δ0 ura3Δ0 snf8::kanMX*	Y.K.O. collection
JW 20 891	MATα *his3Δ1 leu2Δ0 lys2Δ0 ura3Δ0 doa4::kanMX*	Y.K.O. collection
JW 22 100	MATα *his3Δ1 leu2Δ0 lys2Δ0 ura3Δ0 bro1::kanMX*	Y.K.O. collection
JW 22 220	MATα *his3Δ1 leu2Δ0 lys2Δ0 ura3Δ0 vps4::kanMX*	Y.K.O. collection
JW 22 777	MATα *his3Δ1 leu2Δ0 lys2Δ0 ura3Δ0 did2::kanMX*	Y.K.O. collection
JW 24 370	MATα *his3Δ1 leu2Δ0 lys2Δ0 ura3Δ0 vps60::kanMX*	Y.K.O. collection
JW 21 424	MATα *his3Δ1 leu2Δ0 lys2Δ0 ura3Δ0 vta1::kanMX*	Y.K.O. collection
JW 23 123	MATα *his3Δ1 leu2Δ0 lys2Δ0 ura3Δ0 chm7::kanMX*	Y.K.O. collection
JW 20 444	MATα *his3Δ1 leu2Δ0 lys2Δ0 ura3Δ0 ist1::kanMX*	Y.K.O. collection
JW 20 124	MATα *his3Δ1 leu2Δ0 lys2Δ0 ura3Δ0 snf7::kanMX*	Y.K.O. collection
JW 21 479	MATα *his3Δ1 leu2Δ0 lys2Δ0 ura3Δ0 vps24::kanMX*	Y.K.O. collection
JW 22 806	MATα *his3Δ1 leu2Δ0 lys2Δ0 ura3Δ0 vps20::kanMX*	Y.K.O. collection
JW 11 560	MATα *his3Δ1 leu2Δ0 lys2Δ0 ura3Δ0 did4::kanMX*	Y.K.O. collection

The pYe plasmids with the galactose inducible, episomal αAβ_42_-linker-GFP isoforms, the similar Aβ_42_-GFP construct without the α-prepro sequence and the control constructs for expressing of either GFP (ev-GFP) or a α-prepro-GFP fusion (ev-αGFP) were described previously (Tong et al., 2001). The p426-GAL vector (Addgene) was used as additional empty vector (ev) control. For a full list of plasmids used in this study see Table 2.

**Table 2:** Plasmids used in this study.

Name	Backbone	Marker	Insert	Source
αAβ_42_wt	pYe-GAL10 2U	*URA3*	*α-prepro-Aβ_42_wt-linker-GFP*	D’Angelo et al., 2013
αAβ_42_arc	pYe-GAL10 2U	*URA3*	*α-prepro-Aβ_42_arc-linker-GFP*	D’Angelo et al., 2013
αAβ_42_G37C	pYe-GAL10 2U	*URA3*	*α-prepro-Aβ_42_G37C-linker-GFP*	D’Angelo et al., 2013
αAβ_42_G37C-HDEL	pYe-GAL10 2U	*URA3*	*α-prepro-Aβ_42_G37C-linker-GFP-HDEL*	Christophe Cullin
αAβ_42_L34T	pYe-GAL10 2U	*URA3*	*α prepro-Aβ_42_L34T-linker-GFP*	D’Angelo et al., 2013
ev-αGFP	pYe-GAL10 2U	*URA3*	*α-prepro-GFP*	D’Angelo et al., 2013
ev-GFP	pYe-GAL10 2U	*URA3*	*GFP*	D’Angelo et al., 2013
Ev	p426-GAL1	*URA3*		Mumberg et al., 1994
pHS12-mCherry	pHS12-ADH1	*LEU2*	*COX4*	Addgene
pYX242-mCherry	pYX242-TPI	*LEU2*	*Kar2_(1-135)_-mCherry-HDEL*	Swinnen et al., 2014

Standard yeast techniques were applied. We used minimal medium containing Yeast Nitrogen Base (YNB) without ammonium sulfate (FORMEDIUM). Supplements were ammonium sulfate (5 g/L; VWR), histidine (100 mg/L; MP Biomedicals), methionine (20 mg/L; Acros Organics), leucine (30 mg/L; FORMEDIUM) and lysine (30 mg/L; FORMEDIUM). Synthetic Drop-Out (SD) medium was used for microscopy and contained YNB with ammonium sulfate and was depleted for either uracil or uracil and leucine (FORMEDIUM). For solid media, 1.5% Difco-agar (BD) was added. Pre-cultures were grown on medium supplemented with 4% glucose and gene expression was induced by washing the cells with medium without sugar followed by transfer to medium supplemented with 2% galactose.

### Synthetic Genetic Array and Suppressor screening

The synthetic genetic array (SGA) screening was essentially performed as previously described in Tong et al. (2001). The query strain (MATα *can1Δ::STE*pr-*HIS5*sp *lyp1Δ his3Δ1 leu2Δ0 met15Δ0 ura3Δ0 LYS2*) expressing αAβ_42_wt-linker-GFP (further designated as αAβ_42_wt), the αAβ_42_G37C-linker-GFP (designated αAβ_42_G37C) or the ev-αGFP was mated with the non-essential deletion mutant array (MATa *target_gene::kanMX4* *his3Δ1 leu2Δ0 met15Δ0 ura3Δ0*) on SD plates lacking uracil. Diploids were selected on SD medium lacking uracil but containing G418 (geneticin). Next, sporulation was induced by plating diploids onto sporulation medium containing G418. Then, haploids were selected in two steps. First, spores were plated onto YNB lacking arginine, lysine, and histidine but containing canavanine and thialysine, which ensures uptake of canavanine. This allowed growth of MATa haploids only. In a second step, the selected haploids were grown on YNB lacking uracil, arginine, lysine, and histidine but containing canavanine, thialysine and G418 to select for haploid knock-out mutants still carrying the αAβ_42_wt, αAβ_42_G37C, or ev-αGFP plasmids. Growth analysis was performed with the ScreenMill software (Dittmar et al., 2010).

In a second so-called suppressor screening, the Euroscarf collection of deletion strains was pooled transformed with αAβ42G37C. Transformants were plated on minimal medium lacking uracil. After incubation, transformants were selected that grew similar as the isogenic wild-type carrying the empty vector control, also when replica plated on SD medium supplemented with casamino acids. Transformants were then used for bar-code PCR sequencing as to identify their corresponding ORF deletion.

### Electron Microscopy

Electron microscopic analysis of yeast cells was done similar as previously described (Lefebvre-Legendre et al., 2005). Briefly, pellets of yeast cells were placed on the surface of a copper EM grid (400 mesh) coated with formvar. Grids were immersed in liquid propane held at -180°C by liquid nitrogen and then transferred in a 4% osmium tetroxide solution in dry acetone at -82°C for 72 h. They were warmed progressively to RT, and washed three times with dry acetone and stained with 1% uranyl acetate. After washing in dry acetone, the grids were infiltrated with araldite (Fluka). Ultra-thin sections were contrasted with lead citrate and observed in an electron microscope (80 kV; 7650; Hitachi) at the EM facility of the Bordeaux Imaging Center.

### Growth Profile Analysis and Spot Assays

Cells were grown under non-inducing conditions in 96-well plates shaking at 30°C in a Multiskan GO or Multiskan FC microplate spectrophotometer (Thermo Scientific) to an OD_595__nm_ or OD_600__nm_, respectively, of 0.5 to 0.9. Cells were washed with minimal medium containing galactose and diluted to an OD_595__nm_ or OD_600__nm_, respectively, of 0.5 after which growth was monitored every 2 h by OD measurement. Four different transformants were taken per experiment and at least three independent experiments were performed. Growth curves were analyzed with GraphPad Prism v7.03, error bars represent standard deviations.

For spot assays, serial dilutions of precultures were spotted on solid medium containing either glucose or galactose and cells were grown at 30°C. The plates were scanned at days 3 to 6.

### Cytometry and Fluorescence Microscopy

To monitor plasma membrane disruption with propidium iodide (PI) staining and the formation of ROS by dihydroethidium (DHE) staining, cells were grown in SD medium lacking uracil and containing 4% glucose. Once in exponential phase, the cells were washed with and re-suspended in SD medium without uracil but containing 2% galactose as to induce expression of αAβ_42_-linker-GFP and control constructs. Flow cytometric analysis was performed on at least four independent transformants with a Guava easyCyte 8HT benchtop flow cytometer (Millipore) after staining with 5 μM PI for 30 min at 30°C and subsequent washing. Data were analyzed with FlowJo v10 and GraphPad Prism 7.03 software packages. Gates were set in FlowJo v10 with single stained ev-αGFP samples. Further statistical analysis was done in GraphPad Prism v7.03. Error bars represent standard deviations and asterisks the significance calculated with an ordinary Two-way ANOVA.

For the FM4-64/CMAC (Thermo Fisher) and Nile Red (Acros) stainings, the cells were grown as described above. After pre-incubating the cells with CAMAC at 30°C for 30 min, FM4-64 stainings were performed as described before (Zabrocki et al., 2005). For the Nile Red staining, the cells were fixed with formaldehyde (4% final concentration) and stained with 2% Nile Red (60 μg/mL stock) for 30 min with shaking at 30°C. Then, the cells were washed twice with PBS and either stored at 4°C or taken immediately for fluorescence microscopy.

For epifluorescence pictures, cells were pre-grown in selective glucose (4%) containing SD medium to exponential phase. After transfer to SD medium containing 2% galactose to induce expression of αAβ_42_-linker-GFP, the cells were grown at 30°C and pictures were taken after different time intervals using a Leica DM4000B or a DMi8 microscope. For Hoechst stainings, the cells were incubated with 20 nM Hoechst 33342 (Sigma-Aldrich) for 10 min. Pictures were deconvolved with Huygens Essential software (v18.04.0p4 64, Scientific Volume Imaging B.V.) and further processed with the standard FIJI software package (v1.51n) (Schindelin et al., 2012).

### Immunological Techniques

Cultures were grown to exponential phase in 4% glucose containing SD medium, transferred to 2% galactose containing medium and grown overnight. Then, three OD units were harvested by centrifugation and protein extracts were prepared by using an alkaline lysis method. The cells were permeabilized with 0.185 M NaOH plus 2% β-mercaptoethanol. After 10 min incubation on ice, trichloroacetic acid (TCA) was added to a final concentration of 5%, followed by an additional 10 min incubation step on ice. Precipitates were collected by centrifugation for 5 min at 13000 *g* and pellets were resuspended in 50 μL of sample buffer (4% sodium dodecyl sulfate, 0.1 M Tris-HCl pH 6.8, 4 mM EDTA, 20% glycerol, 2% β-mercaptoethanol, and 0.02% bromophenol blue) plus 25 μL of 1 M Tris-Base. Samples were separated by standard SDS-PAGE on 12% polyacrylamide gels and further analyzed using standard Western blotting techniques. An anti-GFP primary antibody (Sigma-Aldrich) and anti-Mouse (GAM)-HRP conjugated secondary antibody (Biorad) were used. The ECL method (SuperSignal West Pico or Femto, Thermo Scientific) was used for detection and visualization of the blots was performed with a UVP Biospectrum^®^ Multispectral Imaging System.

## Results

### αAβ_42_-Linker-GFP Is Toxic in an AD Yeast Model

**FIGURE 1:**
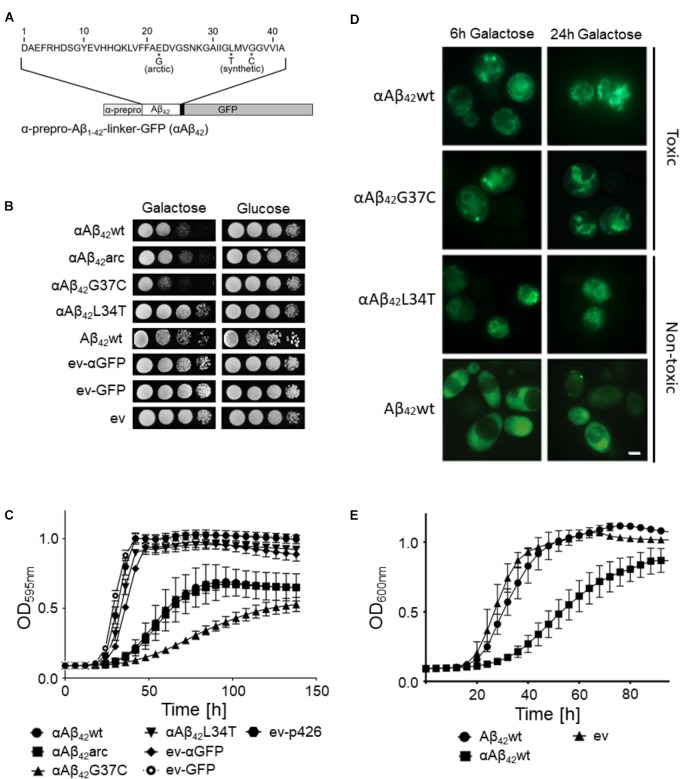
Toxicity profiles of wild-type or mutant αAβ_42_ in the haploid *Saccharomyces cerevisiae* strains BY4741. **(A)** Schematic representation of the α-prepro-Aβ_42_-linker-GFP construct with indication of the mutants used in this study. **(B)** Spot assays under inducing (galactose) and repressing (glucose) conditions and **(C)** growth profiles under inducing conditions of the BY4741 wild-type strain transformed with constructs allowing for expression of αAβ_42_wt, αAβ_42_arc, αAβ_42_G37C, αAβ_42_L34T, Aβ_42_wt and control vectors (ev-αGFP, ev-GFP, ev) as indicated. Error bars in the growth profiles represent the standard deviation of at least four independent transformants. **(D)** Fluorescence microscopy pictures show localization of α-prepro fused Aβ_42_ mutants to the ER 6 and 24 h after induction on galactose-containing medium. 24 h after induction of gene expression the toxic αAβ_42_ isoforms show a more “patchy” pattern while the non-toxic αAβ_42_L34T still localizes at the ER. The Aβ_42_wt construct lacking the α-prepro sequence is seen in the cytoplasm. The scale bar represents 2 μm. **(E)** Growth profiles of the BY4741 wild-type strain transformed with an empty vector (ev) or constructs allowing for expression of αAβ_42_wt and Aβ_42_wt.

To date, the exact molecular basis of how Aβ_42_ impacts cell functions remains largely elusive. To address this question, we used a yeast model transformed with plasmids carrying the galactose-inducible *GAL10* promoter to control expression of wild-type or mutant Aβ_42_ that is N-terminally fused to the α-prepro sequence and C-terminally to a linker and GFP (in this paper referred to as αAβ_42_; Figure 1A) (D’Angelo et al., 2013; Vignaud et al., 2013). Besides the wild-type αAβ_42_ (αAβ_42_wt), we additionally expressed the clinical E22G arctic mutant (αAβ_42_arc) that is associated to a familial form of AD as well as the previously described synthetic mutants αAβ_42_G37C and αAβ_42_L34T (Figure 1A) (D’Angelo et al., 2013; Vignaud et al., 2013). We used three control vectors, i.e., an empty vector allowing for the expression of an α-prepro fused GFP (ev-αGFP), GFP alone (ev-GFP) or an empty vector just carrying the galactose promotor (ev). To disperse concerns about the processing efficacy of the α-prepro factor in the Golgi system, we tested both the BY4741 MATa and BY4742 MATα strains with all control vectors and the aforementioned wild-type and mutant αAβ_42_-linker-GFP constructs (Figures 1B–D and Supplementary Figure S1). Albeit the strains transformed with the ev-αGFP control grew somewhat slower than those transformed with ev-GFP or ev (Figures 1B,C and Supplementary Figure S1A), both spot assays and growth analysis in liquid medium confirmed that wild-type or mutant αAβ_42_ instigated a significantly higher level of toxicity that was similar in the BY4741 and the BY4742 strains (Figures 1B,C and Supplementary Figure S1). The synthetic αAβ_42_G37C mutant was the most toxic followed by αAβ_42_wt and αAβ_42_arc. The synthetic αAβ_42_L34T mutant, on the other hand, did not yield a toxic phenotype and these transformants grew similar as those expressing ev-αGFP, thereby confirming previously reported data (Vignaud et al., 2013).

Fluorescence microscopy showed that all αAβ_42_ constructs clearly stained the perinuclear ER and to a lesser extent the cortical ER and that particularly the cells expressing the toxic αAβ_42_ forms often displayed ER-associated foci and filamentous structures (Figure 1D). These were previously believed to be vesicles (D’Angelo et al., 2013), but recent studies suggests that these may as well-represent ER-aggregates or clustering of ER membranes, both indicative for ER stress (Varadarajan et al., 2012, 2013; Vevea et al., 2015). In contrast, when the Aβ_42_wt was expressed from a construct that lacks the α-prepro sequence, the GFP fusion was mainly found to be distributed in the cytoplasm though some cells presented foci after prolonged induction (Figure 1D). Despite of these foci, the expression of the Aβ_42_wt construct without the α-prepro sequence only triggered a small growth retardation (Figures 1B,E). This nicely demonstrates that the processing in the ER/Golgi system is required to unleash the full toxic capacity of αAβ_42_.

### Retention of αAβ_42_ in the ER Diminishes Its Toxicity

**FIGURE 2:**
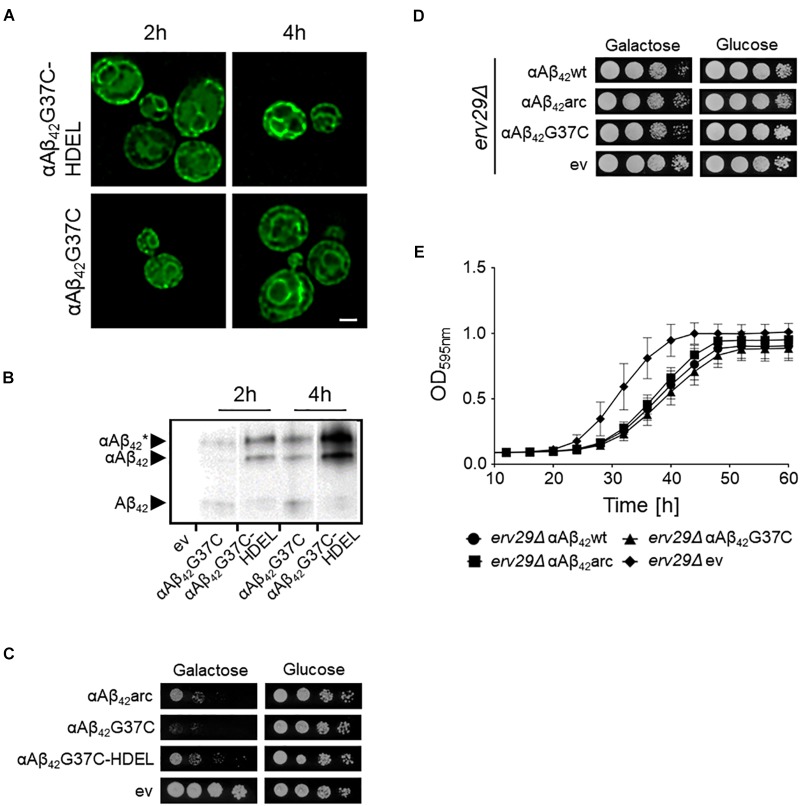
Retention of αAβ_42_ in the ER and the consequence for toxicity. **(A)** Fluorescence microscopy pictures showing αAβ_42_G37C and αAβ_42_G37C-HDEL localization at the perinuclear and cortical ER. Scale bar represents 2 μm. **(B)** Western blot analysis of total protein extracts obtained from cells expressing either αAβ_42_G37C and αAβ_42_G37C-HDEL. The different processing isoforms are indicated, i.e., αAβ_42_^∗^ indicates glycosylated αAβ_42_, αAβ_42_ indicates un-glycosylated αAβ_42_ and Aβ_42_ indicates fully processed form where the α-prepro sequence is cleaved off. Spot assays under inductive (galactose) and repressive (glucose) conditions of wild-type **(C)** or *erv29Δ* **(D)** cells transformed with the empty vector (ev) or constructs allowing for expression of αAβ_42_wt, αAβ_42_arc, αAβ_42_G37C, or αAβ_42_G37C-HDEL as indicated. **(E)** Growth profiles of *erv29Δ* transformed with the empty vector (ev) or constructs allowing for expression of αAβ_42_wt, αAβ_42_arc, or αAβ_42_G37C when grown on galactose-containing medium.

Previously, we reported that the processing of the αAβ_42_-linker-GFP fusion constructs in the ER/Golgi system yields three distinct isoforms when performing Western blot analysis, i.e., the α-prepro precursor (41 kDa), the glycosylated precursor (50 kDa), and the matured Aβ_42_-linker-GFP form (34 kDa) (D’Angelo et al., 2013; Vignaud et al., 2013). Given that the latter is shuttled into the secretory pathway, we wondered if retention of the αAβ_42_-linker-GFP fusion in the endoplasmic reticulum (ER) would affect its toxic capacity. To this end, we introduced a HDEL retention signal at the C-terminal end of the construct. The yeast HDEL sequence is equivalent to the mammalian KDEL retention signal that shuttles the KDEL containing proteins back to the ER lumen (Dean and Pelham, 1990; Lewis and Pelham, 1990; Ruan et al., 2017). Consistently, microscopic analysis demonstrated that while both αAβ_42_G37C and αAβ_42_G37C-HDEL are equally present at the perinuclear ER, the latter accumulated more in the peripheral cortical ER (Figure 2A). Also, Western blot analysis showed that in case of expression of αAβ_42_G37C-HDEL, both the non-processed αAβ_42_G37C precursor as well as the glycosylated version accumulated, while the fully processed Aβ_42_G37C was significantly reduced (Figure 2B). Next, a growth analysis was performed. This revealed that the strain expressing αAβ_42_G37C-HDEL construct grew much better than that with αAβ_42_G37C, thereby displaying a level of toxicity comparable to a strain with the αAβ_42_arc mutant (Figure 2C).

In order to confirm these results with a different approach, we expressed αAβ_42_wt, αAβ_42_arc, as well as the super-toxic αAβ_42_G37C mutant in a BY4741 strain deleted for *ERV29*, which encodes a transmembrane factor involved in COP-II dependent vesicle formation and in trafficking of the α-prepro factor from the ER to the Golgi apparatus (Belden and Barlowe, 2001). Upon deletion of *ERV29*, αAβ_42_ gets stuck in the ER and cannot transit to the Golgi. Strikingly, cytotoxicity of all tested mutants was indeed significantly diminished in this *erv29*Δ strain and even the super-toxic αAβ_42_G37C mutant almost completely lost its toxic power (Figures 2D,E). Thus, the data above confirm that αAβ_42_ needs to be fully processed and exit the ER in order to gain its full toxic potential.

### αAβ_42_ Affects Mitochondrial Functioning

**FIGURE 3:**
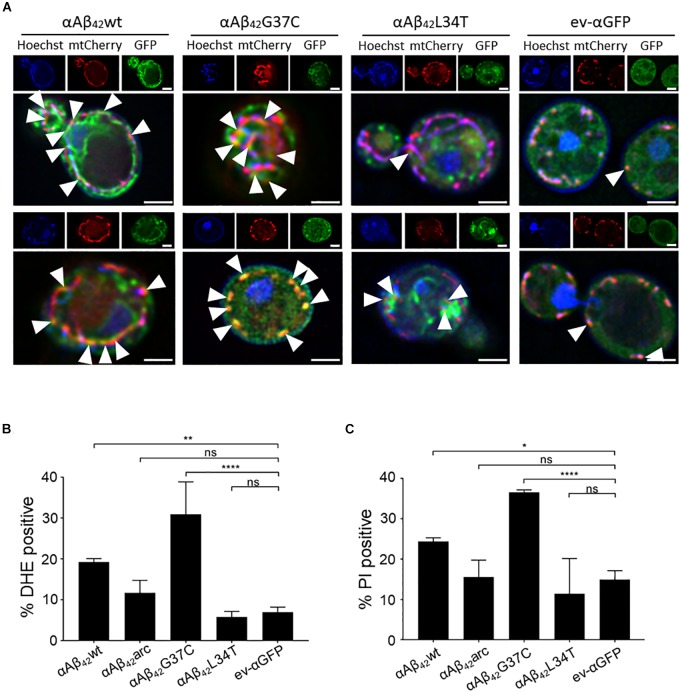
αAβ_42_ induces mitochondrial dysfunction and cell demise. **(A)** Fluorescence microscopy pictures suggesting partial co-localization of toxic αAβ_42_ but not of the non-toxic αAβ_42_L34T nor of the αGFP control (green) with mitochondria (red) in wild-type cells. Hoechst staining (blue) shows mitochondrial as well as nuclear DNA. The white arrowheads indicate sites of co-localization between αAβ_42_ and mitochondria. Two single cells shown per strain. Scale bars represent 2 μm. Percentage of wild-type yeast cells expressing wild-type or mutant αAβ_42_ and a control stained with DHE as a marker for ROS-formation **(B)** or PI as a marker for plasma membrane integrity **(C)**. ^∗^*P* ≤ 0.05; ^∗∗^*P* ≤ 0.01; ^∗∗∗∗^*P* ≤ 0.0001; ^ns^*P* > 0.05.

Since αAβ_42_ confers toxicity by entering the secretory pathway, we wanted to know more about the targets and processes being affected. Therefore, we first focused on mitochondria given that previous studies have associated Aβ_42_ to mitochondrial dysfunction in yeast (Chen and Petranovic, 2015; Chen et al., 2017; Hu et al., 2018). To this end, we co-expressed αAβ_42_wt, αAβ_42_arc, αAβ_42_G37C, αAβ_42_L34T or the ev-αGFP together with a mCherry labeled mitochondrial marker Cox4 in wild-type cells. As illustrated in Figure 3A, this suggests a toxicity dependent co-localization since αAβ_42_wt and even more αAβ_42_G37C seem to partially co-localize with mitochondria. We then performed a DHE staining to estimate the ROS levels as marker for mitochondrial dysfunction and a PI staining to monitor the amount of cells with disrupted plasma membranes as marker for cell demise (Figures 3B,C and Supplementary Figure S2). As expected, and consistent with previously reported data (Chen and Petranovic, 2015; Chen et al., 2017), also these aspects correlated to the observed αAβ_42_ instigated toxicity. Although our data on co-localization suggest that αAβ_42_ may directly interfere with mitochondrial functioning, we cannot exclude that these effects on mitochondrial function or morphology are indirect, for instance due alterations in ER-mitochondrial communication via ERMES contact sites.

### Identification of Additional Processes Underlying αAβ_42_ Toxicity

To decipher which additional processes sustain the αAβ_42_ toxicity, we performed two unbiased genetic screens. In a first screening setup, we aimed to identify alleviators of Aβ_42_G37C toxicity. Therefore, we transformed a pooled Euroscarf knock-out (KO) library with the αAβ_42_G37C-linker-GFP construct and looked for transformants that grew similar as the empty vector control. Out of 90,000 transformants obtained when plated on repressive glucose containing medium, only 465 were able to form visible colonies after 72 h on galactose-containing medium where the expression of the super-toxic Aβ_42_ mutant is induced. Each colony was then plated on SD medium supplemented with casamino acids to manually confirm the suppressive effect on αAβ_42_ toxicity. Of the 465 initial clones, 268 were still able to grow. Finally, we characterized each KO strain by sequencing the PCR amplicon of its bar-code region. This led us to identify 113 different KO strains. In the second screening setup, we performed an unbiased SGA analysis with the full gene knock-out library using either αAβ_42_wt, the super-toxic αAβ_42_G37C mutant and the ev-GFP control. The additional use of the empty vector control and αAβ_42_wt, which displays a more moderate toxicity phenotype, allowed for more complete results since not only suppressors but also toxicity aggravators could be scored. With this SGA screen, we identified 87 additional KO strains that modulated αAβ_42_ toxicity, one of which contains a deletion in the overlapping genes *INP52/RRT16*. Finally, the 200 KO strains that were identified by either one of the screening procedures (Table 3 and Supplementary Table S1) were also transformed individually with a construct allowing for the expression of αAβ_42_arc, which has an intermediate toxicity similar to that of αAβ_42_wt and thus is again ideal to provide confirmation of both aggravators and suppressors of toxicity (D’Angelo et al., 2013). This provided an independent confirmation that all the KO strains selected in one of the two genetic screens are indeed involved in modulating αAβ_42_arc toxicity. For each of the KO strains, the cell density under galactose inducing conditions was monitored allowing to rank KO strains from 0 to 5 depending on the growth capacity in comparison to the wild-type strain transformed αAβ_42_arc. For 29 KO strains, growth was improved strongly (scored as 5), for 18 KO strains growth was clearly improved (scored as 4) and for 129 KO strains growth was only slightly better (scored as 3). On the other hand, we also identified 2 KO strains in which αAβ_42_arc toxicity was strongly enhanced (scored as “0”) and 17 KO strains that enhanced αAβ_42_arc toxicity moderately (scored as 1) or weakly (scored as 2) (Table 3 and Supplementary Table S1). Next, a gene ontology (GO) analysis using the SGD Gene Ontology Slim Mapper^1^ allowed to sort the KO strains into functional categories depending on the gene deleted (Table 3). These included, amongst others, cytoskeleton organization and endocytosis, protein sorting and trafficking, protein ubiquitination, plasma membrane transport, cell cycle, translation, and transcription.

**Table 3:** αAβ_42_ toxicity modifiers.

GO term	Enhancers	Suppressors
**Biological process unknown**	**FMP41** (2), **MTC3** (2), YBR113W (2), YHL005C (2), YJL169W (2)	GL081W (3), ICY1 (3), IRC10 (3), MRX11 (3), NBA1 (3), NRP1 (3), PHM7 (3), PRM9 (3), RRT16 (3), TDA8 (3), URN1 (3), YBR209W (3), YBR284W (3), YCR085W (3), YCR099C (3), YDR042C (3), YER067C-A (3), YER186C (3), YGL101W (3), YGR259C (3), YIL014C-A (3), YKL066W (3), YL043W (3), YLR042C (3), YLR279W (3), **YLR283W** (3), YML096W (3), YML119W (3), YMR102C (3), YMR153C-A (3), YMR160W (3), YMR178W (3), YMR244W (3), YMR316C-A (3), YMR317W (3), YNL146W (3), YOR263C (3), YPL257W (3), YGL109W (4), YJL007C (4), YMR114C (4), YNL338W (4), YNR071C (4), YGL072C (5), YLR149C (5), YML084W (5), YMR103C (5), YOR296W (5), YPL247C (5)
**Cell cycle**	**END3** (2), **RIM8** (2), **CCR4** (2)	**CLN1** (3), **DIA2** (3), **FYV5** (3), **GAS2** (3), **MAM1** (3), **NFI1** (3), **PCL1** (3), **PEA2** (3), **REC114** (3), **PRM3** (3), **SMK1** (3), **SRC1** (3), **SSO2** (3), **SSP1** (3), **TEC1** (3), **TEP1** (3), **TOF1** (3), **YOR338W** (3), **BDF2** (4), **ITC1** (4), **REI1** (4), **XRN1** (4), **ADA2** (5), **CLB3** (5), **CSM4** (5), **STE24** (5), **YHP1** (5), **ZDS1** (5)
**Cell morphology**	**END3** (2)	**GAS2** (3), **GPD2** (3), **PEA2** (3), **SMK1** (3), **SSP1** (3), **TEP1** (3), **REI1** (4)
**Cytoskeleton**	SAC6 (0), **BEM2** (2), **END3** (2), **RVS167** (0), **SLA1** (2) TDA2 (2)	**ABP1** (3), **PCL1** (3), **PEA2** (3), **SLM2** (3), **WHI2** (3), **CLB3** (5), **PFD1** (5)
**DNA**	**CCR4** (2), **SLX8** (2)	**ADA2** (5), **CSM4** (5), **DIA2** (3), HCS1 (3), **RAD34** (3), **REC114** (3), **RPB9** (3), **SLX5** (3), **TEC1** (3), **TOF1** (3)
**Mitochondria**	**MTC3** (2), **FMP41** (2), **RTG1** (2), **SAM37** (2)	**HEM25** (3), MRX5 (3), **AIM25** (3), **YLR283W** (3), **GUF1** (3), **AIF1** (3), **ODC1** (3), **MDL2** (3), **YME1** (3), **SUE1** (3), **PKP1** (4), **FMP25** (4), **RSM25** (5), **FMP30** (5)
**Metabolism**	**TDA9** (2)	ALD2 (3), ALD3 (3), CPA1 (3), **FAA1** (3), FAA3 (3), FAU1 (3), **GPD2** (3), LEU4 (3), NMA1 (3), SNO2 (3), SUR1 (3), YDC1 (3), IGD1 (4), **FMP30** (5), HIS1 (5), **PSK2** (5), SPE2 (5)
**Organelle organization**	**SAM37** (2), **RIM8** (2)	**ABP1** (3), **AIM25** (3), **ATG1** (3), ATG2 (3), **ATG20** (3), DID2 (3), **DJP1** (3), IWR1 (3), **MAM1** (3), **NGR1** (3), **PEA2** (3), **PEX6** (3), **PRM3** (3), **REC114** (3), SPC2 (3), **SRC1** (3), **SSO2** (3)**TOF1** (3), VPS68 (3), **WHI2** (3), **YME1** (3), **BDF2** (4), **FMP25** (4), **ADA2** (5), **CLB3** (5), **CSM4** (5) **SCD6** (5)
**Other**	**BEM2** (2), **END3** (2), **RIM8** (2), **RTG1** (2)	ADH6 (3), APE2 (3), ARF2 (3), **DIA2** (3), FDO1 (3), **FYV5** (3), IGO2 (3), **ODC1** (3), OXP1 (3), **PEX6** (3), PML39 (3), **SLM2** (3), **GAS2** (3), **SMK1** (3), **SSO2** (3), **SSP1** (3), **SUE1** (3), **TEC1** (3), **TEP1** (3), TVP18 (3), YML082W (3), **YOR338W** (3), ECM4 (4), PDE2 (4), YGR111W (4)
**Protein modification**	**SNF7** (1), HPM1 (2), **PKR1** (2), **RIM8** (2), **SAM37** (2), **SLA1** (2), **SLX8** (2), **UMP1** (2), **VPS24** (2)	**ABP1** (3), **ATG1** (3), **CLN1** (3), CPS1 (3), CUL3 (3), CUR1 (3), **DIA2** (3), **NFI1** (3), **HRT3** (3), **MAM1** (3), **PCL1** (3), **SLX5** (3), **SMK1** (3), SPC2 (3), **SSP1** (3), TUL1 (3), **YME1** (3), **FMP25** (4), **PKP1** (4), **XRN1** (4), **ADA2** (5), **CLB3** (5), **PFD1** (5), **PSK2** (5), **STE24** (5)
**RNA**	**CCR4** (2)	DEG1 (3), HIT1 (4), NGL2 (3), **RPB4** (3), RPS8A (3), **XRN1** (4), CBC2 (5), MSL1 (5), SNT309 (5), **TSR3** (5)
**Stress response**	**SNF7** (1), **PKR1** (2), **PMP3** (2), **RTG1** (2), **RVS167** (0), **SLX8** (2), **UMP1** (2)	**AIF1** (3), **AIM25** (3), CUR1 (3), **FYV5** (3), HYR1 (3), **MDL2** (3), MIG1 (3), **PEA2** (3), **RAD34** (3), **RPB4** (3), **RPB9** (3), **SLX5** (3), SMF3 (3), **TOF1** (3), YCR102C (3), **ITC1** (4), TRK1 (5), **WHI2** (3), **ADA2 (5), YAR1 (5)**
**Transcription**	**CCR4** (2), **RTG1** (2), **TDA9** (2)	CAF120 (3), FUI1 (3), GAT2 (3), **ITC1** (4), MIG1 (3), **RPB4** (3), **RPB9** (3), **TEC1** (3), **YOR338W** (3), FZF1 (4), STP1 (4), **XRN1** (4), **ADA2** (5), MBF1 (5), **PFD1** (5), **YHP1** (5)
**Translation**	RPS7A (2)	**GUF1** (3), **NGR1** (3), **RPB4** (3), RPS7B (3), RPS8A (3), HIT1 (4), **REI1** (4), **PSK2** (5), RPL2B (5), **RSM25** (5), **SCD6** (5), **TSR3** (5), **YAR1 (5)**
**Transport (not vesicular)**	**SNF7** (1), **PMP3** (2), **SAM37** (2), VPS24 (2)	ATO3 (3), **DJP1** (3), **FAA1** (3), FUI1 (3), GFD1 (5), **HEM25** (3), IWR1 (3), **MDL2** (3), MSN5 (3), NRT1 (3), **PEX6** (3), PUT4 (3), **RPB4** (3), SMF3 (3), **YME1** (3), YOL163W (3), FZF1 (4), **REI1** (4), **YAR1 (5)**, **ZDS1** (5)
**Vesicles/trafficking**	**RVS167** (0), **SNF7** (1), **END3 (2), RIM8** (2), **SLA1** (2), **VPS24** (2)	**ATG20** (3), DID2 (3), EMP24 (3), **PRM3** (3), **SLM2** (3), **SSO2** (3), VPS68 (3), **WHI2** (3), YPT31 (3), SLM6 (4), ERV29 (5)

*Enhancers and suppressors of the αAβ_*42*_ instigated toxicity are shown. Genes are sorted according to the Gene Ontology (GO) terms. Category “Mitochondria” was manually added according to GO terms list. Genes that occur in more than 1 GO-term category are in bold. The numbers between brackets refer to the growth scores ranging from 0 (worst growth) to 5 (best growth). See main text for details*.

Interestingly, among the many KO strains that alleviated αAβ_42_arc toxicity we found not only the strain deleted for *ERV29*, which is in line with the data described above, but also strains lacking other genes that impact on ER/Golgi functioning and traffic, such as the *SPC2* encoded subunit of the peptidase complex, which cleaves the signal sequence from proteins targeted to the ER, *EMP24*, which encodes a component of the p24 complex that mediates ER-to-Golgi transport of GPI anchored proteins, or *ARF2* and *YPT31*, which both encode GTPases required for intra-Golgi traffic. Also found were several strains lacking genes encoding mitochondrial functions and this included *AIF1* that codes for the mitochondrial cell death effector, indicative that αAβ_42_ actively induces programmed cell death pathways. Furthermore, the fact that we retrieved the KO strains for *ATG1, ATG2*, and *ATG20* suggests that αAβ_42_ may overstimulate the autophagy and the cytoplasm-to-vacuole targeting pathways (Kim and Klionsky, 2000; Nice et al., 2002). Finally, it was recently shown by transcriptome analysis that Aβ_42_ impacts on lipid metabolism with *INO1* being most significantly upregulated (Chen et al., 2017). We found the KO strain lacking *ITC1* to reduce αAβ_42_ toxicity. *ITC1* encodes a subunit of ATP-dependent Isw2p-Itc1p chromatin remodeling complex that is required for repression of *INO1*. In addition, also KO strains lacking genes involved sphingolipid and ceramide metabolism were retrieved, i.e., *FAA1, SUR1, YDC1*. Overall, these data confirm that the noxious effect of αAβ_42_ is associated to changes in lipid metabolism.

**FIGURE 4:**
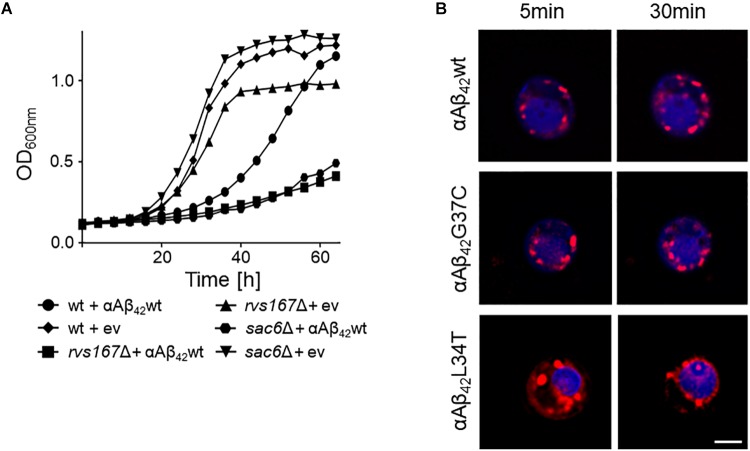
Interference of αAβ_42_ with endocytosis. **(A)** Growth profiles of wild-type cells and *rvs167Δ* or *sac6Δ* cells transformed with the empty vector (ev) or a construct allowing for expression of αAβ_42_wt when grown on galactose-containing medium. **(B)** Fluorescence microscopy pictures of strains expressing the toxic αAβ_42_wt and αAβ_42_G37C or the non-toxic αAβ_42_L34T stained with the endocytosis tracker FM4-64 (red) and CMAC (blue), a dye to stain the vacuolar lumen. The scale bar represents 2 μm.

The KO strains that aggravated αAβ_42_ toxicity were often missing functions associated to maintenance and organization of the actin cytoskeleton, endocytosis and the multivesicular body (MVB) pathway. The two strains with the strongest enhanced toxicity were those carrying a deletion of *SAC6* or *RVS167*. Sac6 is an actin-bundling protein that is required for endocytosis (Penalver et al., 1997; Gheorghe et al., 2008). Rvs167 is a homolog of mammalian amphiphysin that interacts with actin as well and that functions in the internalization step of endocytosis (Lombardi and Riezman, 2001). As illustrated for the expression of αAβ_42_wt, we indeed observed a similar severe growth phenotype in both the *sac6Δ* and *rvs167Δ* mutants as compared to the isogenic wild-type strain (Figure 4A). To further confirm the link between endocytosis and αAβ_42_ toxicity, we also monitored the uptake of the endocytosis tracker FM4-64 by wild-type cells expressing either the toxic αAβ_42_wt and αAβ_42_G37C and non-toxic αAβ_42_L34T isoforms after 4 h induction on galactose-containing medium. As shown, FM4-64 already stained the vacuolar membrane within 30 min in cells expressing the non-toxic construct while no, or only a minimal staining of the vacuolar membrane was observed in cells expressing the toxic αAβ_42_ species, even not after 60 min of incubation (Figure 4B). This demonstrates that the latter have a direct impact on the endocytic process.

### αAβ_42_ Enhances the Occurrence of Plasma Membrane Lesions and Formation of Lipid Droplets

**FIGURE 5:**
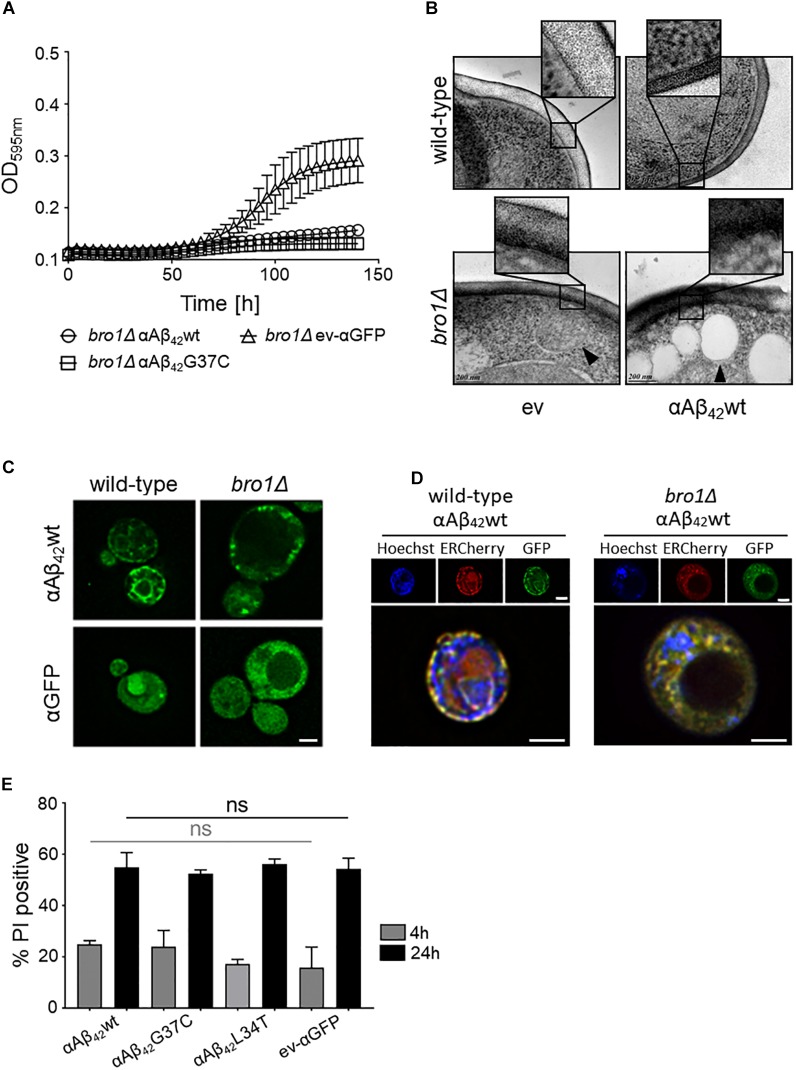
The role of Bro1 for membrane lesion repair. **(A)** Growth profile on galactose-containing medium of a strain deleted for the ESCRT-III accessory factor *BRO1* transformed with an empty vector (ev-αGFP) or constructs allowing for expression of αAβ_42_wt or αAβ_42_G37C. Cryo-EM pictures **(B)** and fluorescence microscopy pictures **(C)** of wild-type and *bro1Δ* cells transformed with an empty vector (ev) or expressing αAβ_42_wt and grown for 6 h on galactose-containing medium. The indents in panel **(B)** zoom in on the plasma membrane and cell wall. The black arrowhead in **(B)** indicates a lipid droplet. Scale bars for cryo-EM pictures represent 200 nm. **(D)** BY4742 wild-type and a *bro1*Δ strains transformed with a plasmids carrying αAβ_42_wt and additionally a plasmid allowing the expression of Kar2_(1-135)_-mCherry-HDEL (ERCherry), a marker for the ER. DNA was stained with Hoechst. Cells were grown in medium allowing for gene expression for 6 h prior to microscopy. Scale bars for fluorescence pictures represent 2 μm. **(E)** PI staining of cells deleted for *BRO1* transformed with constructs allowing for expression of αAβ_42_wt, αAβ_42_L34T, αAβ_42_G37C, or αGFP after 4 or 24 h growth on galactose-containing medium. Error bars represent standard deviations of at least four independent transformants.

Closely connected to endocytosis, we noticed that our screening retrieved some ESCRT components, which function in the MVB pathway that is required for the turnover of plasma membrane proteins and lipids. The first step in MVB formation is the binding of ESCRT-0 (Hse1 and Vps27) and ESCRT-I (involving Vps28, Mvb12, Srn2, and Stp22) to ubiquitinated MVB cargoes. Next, the ESCRT-II complex (involving Vps25, Vps36, and Snf8) mediates the recruitment of ESCRT-III accessory factors (Bro1 and Doa4), which in turn loads general ESCRT-III factors (Vps20, Snf7, Did4, Chm7, Ist1, and Vps24) to direct the continued sorting of cargoes into invaginating vesicles during MVB formation. ESCRT-III dissociation factors (Vps4, Vps60, Did2, and Vta1) mediate the release and recycling of all involved factors (Hurley, 2010; Babst, 2011). All the 21 corresponding genes are not essential and could therefore be tested for their implications in αAβ_42_ toxicity. After transformation of the corresponding KO strains with either αAβ_42_wt, αAβ_42_G37C or the ev-αGFP control construct, we evaluated the impact of the deletions on αAβ_42_ toxicity through spot assays. This revealed that for several components of the MVB pathway their deletion significantly aggravated αAβ_42_ toxicity. This included the ESCRTIII components Did4, the ESCRTIII accessory components Bro1 and Doa4 as well as the ESCRTIII dissociation mediator Vps4 (Supplementary Figures S3, S4). Recent studies in mammalian cells have demonstrated that besides its role in the MVB pathway, ESCRT plays key roles in a variety of other processes, including membrane lesion repair (Jimenez et al., 2014; Sundquist and Ullman, 2015; Campsteijn et al., 2016; Denais et al., 2016; Raab et al., 2016). It is well-established that Aβ_42_ can introduce membrane lesions and different non-excluding mechanisms were proposed, including membrane lipid interaction, alterations in membrane fluidity, pore formation, or lipid oxidation (Eckert et al., 2000; Muller et al., 2001; Ambroggio et al., 2005; Rauk, 2008; Axelsen et al., 2011; Viola and Klein, 2015). We speculated that αAβ_42_ would also trigger membrane lesions in our yeast system. To test this, we analyzed the plasma membrane integrity of wild-type and *bro1*Δ cells by cryo-EM. We chose the *bro1Δ* strain because here αAβ_42_wt and αAβ_42_G37C was almost lethal (Figure 5A) and because Bro1 is the yeast ortholog of human Alix, a proposed biomarker for AD (Sun et al., 2015). Consistent with our hypothesis, the cryo-EM study showed that the plasma membrane of *bro1*Δ cells expressing αAβ_42_wt seemed heavily corrugated while the *bro1*Δ cells transformed with empty vector displayed a more modest phenotype. In the wild-type strain, the plasma membrane remained ostensibly smooth even upon expression of αAβ_42_wt (Figure 5B). However, although we did not quantify, the observed membrane corrugating effects of specifically the *bro1*Δ strain expressing αAβ_42_wt are strikingly obvious. Moreover, when compared to the wild-type cells, the ER morphology in the *bro1*Δ mutant was completely different and appeared to be deteriorated. Indeed, only a minimal perinuclear and cortical ER was detected and the cells displayed the αAβ_42_-linker-GFP fusion mostly in filamentous structures and foci (Figures 5C,D). Thus, expression of αAβ_42_wt seems to dramatically affect all membranous structures evidencing that Bro1, and by extension the ESCRT system, is absolutely required for the repair of membrane lesions induced by αAβ_42_. In line with this essential requirement of ESCRT, we observed that while still seeing a tendency of increased PI uptake after 4 h induction for those *bro1*Δ cells expressing toxic αAβ_42_, a maximal PI uptake in all *bro1*Δ transformants is seen after 24 h, even for those strains expressing the non-toxic αAβ_42_L34T mutant or the ev-αGFP control (Figure 5E).

**FIGURE 6:**
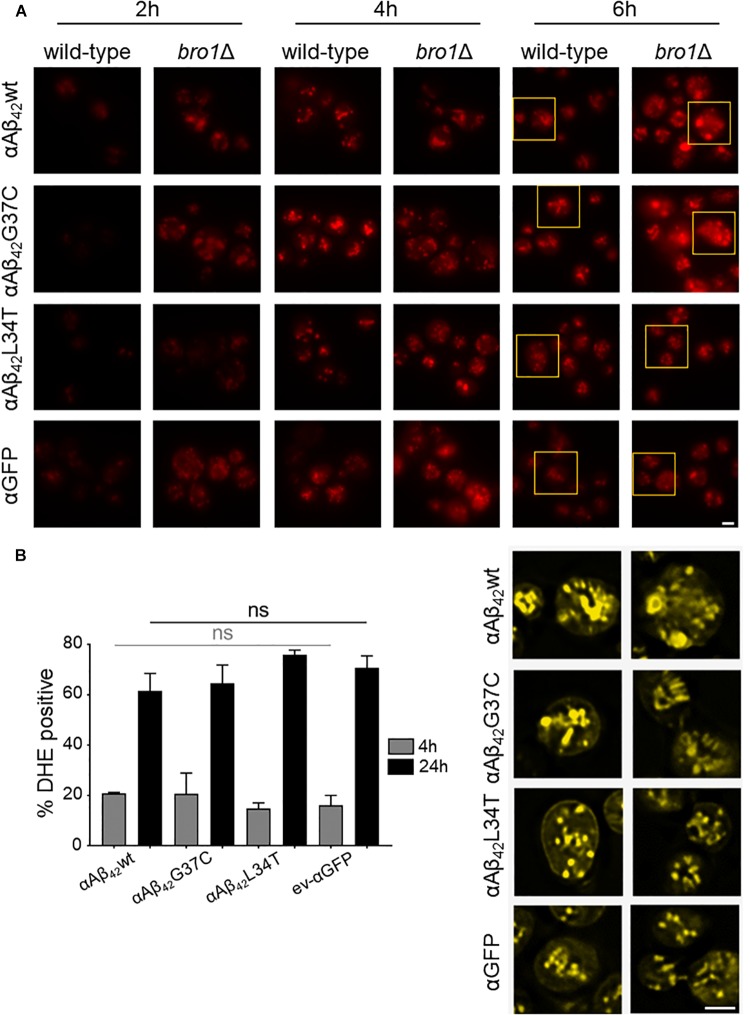
αAβ_42_ instigated lipid droplet biogenesis. **(A)** Nile Red stainings (top panel), a marker for lipid droplets, of wild-type and the *bro1Δ* cells expressing αAβ_42_wt, αAβ_42_G37C, or αAβ_42_L34T or αGFP after 2, 4, or 6 h growth on galactose-containing medium. The bottom panel (yellow) shows magnifications and deconvolved parts of the top-panel pictures at time point of 6 h. Note that due to deconvolution the picture intensities are enhanced. Scale bars represent 2 μm. **(B)** DHE staining of cells deleted for *BRO1* transformed with constructs allowing for expression of αAβ_42_wt, αAβ_42_L34T, αAβ_42_G37C, or αGFP after 4 or 24 h growth on galactose-containing medium. Error bars represent standard deviations of at least four independent transformants.

The cryo-EM study also pointed to the formation of cortical vesicle-like structures, which we believe may correspond to lipid droplets. Since previous studies have shown that the formation of lipid droplets denotes an adaptive response to a chronic lipid imbalance (Vevea et al., 2015; Garcia et al., 2018), which is likely to occur in *bro1Δ* strain because of hampered MVB formation and lipid turnover, we decided to perform a Nile Red staining to visualize the droplets. While during the first hours of induction on galactose-containing medium we observed an overall enhanced lipid droplet biogenesis in all *bro1*Δ strains as compared to the respective wild-type strains, the increase was persistent and especially more dense droplets were seen with *bro1*Δ cells expressing αAβ_42_G37C or αAβ_42_wt (Figure 6A). This observation confirms previously reported data on an increased lipid droplet load in an Aβ_42_ wild-type strain (Chen et al., 2017).

The perturbation of lipid homeostasis has also been linked to defects in the ER and mitochondria (Vevea et al., 2015), and the latter incited us to monitor the level of ROS in the *bro1*Δ strains. As shown, and comparable to the PI uptake, we found similar high levels of ROS in all strains tested indicative that this is a characteristic mainly associated to the deletion of *BRO1* itself (Figure 6B).

## Discussion

During the past decades, several studies validated the use of yeast to decipher the pathobiology underlying a variety of human disorders. Especially for degenerative protein folding diseases, like Huntington’s, Parkinson’s, or Alzheimer’s disease, this led to the discovery of processes and molecular pathways contributing to cell demise (Winderickx et al., 2008; Franssens et al., 2010; Swinnen et al., 2011; Porzoor and Macreadie, 2013; Tenreiro et al., 2013, 2017; Chen and Petranovic, 2016; Jiang et al., 2016; Verduyckt et al., 2016; Fruhmann et al., 2017). The insight gained from these studies were not only relevant in the context of disease, but they also clarified fundamental aspects on how a cell manages to maintain proteostasis and the consequences in case this system fails. In this paper, we used a previously reported model to study the repercussions when the APP-derived peptide Aβ_42_ is expressed in yeast (D’Angelo et al., 2013; Vignaud et al., 2013). Though this model makes use of a GFP fusion, the use of the super-toxic Aβ_42_G37C and the non-toxic Aβ_42_L34T mutants clearly demonstrated that the properties of the GFP fusion are dictated by the Aβ_42_ peptide moiety. Furthermore, by comparing constructs with or without an N-terminal fusion with the α-prepro sequence and conditions that retain the α-prepro in the ER, it became obvious that the processing in the ER/Golgi system and the subsequent shuttling into the secretory pathway is essential to unleash the full toxic capacity of the Aβ_42_-linker-GFP fusion. Our data also contradict the argument that the αAβ_42_ instigated toxicity would simply be due to an overload of the ER/Golgi processing system because both wild-type and mutant αAβ_42_ are processed in the same manner (D’Angelo et al., 2013; Vignaud et al., 2013) and no toxicity is seen in case of expression of mutant αAβ_42_L34T.

The use of our yeast model allowed us to confirm some data previously reported. This included the impact of αAβ_42_ on endocytosis (Treusch et al., 2011; D’Angelo et al., 2013), where we now show that strains with a deletion of *SAC6* or *RVS167* display an increased αAβ_42_ toxicity, as well as the impact on mitochondrial functioning (D’Angelo et al., 2013; Chen and Petranovic, 2015; Chen et al., 2017), which we illustrated by co-localization studies and the observation that αAβ_42_ enhances ROS formation. These data are relevant because, indeed, changes in endocytic capacity and mitochondrial dysfunction are typically seen in the pathogenesis of AD and have been observed in other AD models as well (Thomas et al., 2011; Wang et al., 2011; Avetisyan et al., 2016; Schreij et al., 2016; Dixit et al., 2017; Shoshan-Barmatz et al., 2018; Xu et al., 2018). However, the observed partial co-localization of toxic αAβ_42_ with mitochondria may either indicate a direct interaction of the peptide with this organelle or it may simply be a reflection of the interaction between the ER and mitochondria through the membrane contact sites known as ERMES (Kornmann et al., 2009). This aspect needs to be analyzed in more detail. Interestingly, mitochondria associated membranes (MAMs), the mammalian counterpart of ERMES, have already been implicated in AD (Area-Gomez and Schon, 2016; Paillusson et al., 2016). Closely related to the observed mitochondrial dysfunction is our observation that deletion of *AIF1*, encoding a cell death effector, has a protective effect on the αAβ_42_ expressing yeast cells. This suggests that αAβ_42_ may induce an apoptotic-like program in yeast, which fits the finding that neuronal cells die through apoptosis in AD (Ohyagi et al., 2005; Shoshan-Barmatz et al., 2018). Our data also show that particularly the expression of the toxic αAβ_42_ isoforms is linked to the formation of ER-associated foci and filamentous structures. Though we did not study these structures in detail and previously believed these to represent vesicles (D’Angelo et al., 2013), it is well-possible that they may in fact be ER aggregates or clustering of ER membranes, which are both indicative for ER stress (Varadarajan et al., 2012, 2013; Vevea et al., 2015). Also ER stress is associated to AD (Gerakis and Hetz, 2018) and several links between ER stress and mitochondrial dysfunction have been proposed in this neurodegenerative disorder (Costa et al., 2012; Barbero-Camps et al., 2014; Erpapazoglou et al., 2017).

One of the most striking observations made in our studies is the role of ESCRT in modulating the αAβ_42_ toxicity. The ESCRT system functions in the MVB pathway and several studies have linked this role of ESCRT to AD. Neurons of AD transgenic mice were shown to display enlarged MVBs as compared to the neurons of wild-type mice, and ESCRT was demonstrated to modulate intracellular Aβ_42_ accumulation by directing APP to lysosomal degradation and by enhancing Aβ_42_ secretion. In addition, ESCRT components were found associated with amyloid plaques in transgenic mice and to granular structures hippocampal neurons of AD diseased human brain (Yamazaki et al., 2010; Edgar et al., 2015; Willen et al., 2017). However, apart from its function in the MVB pathway, ESCRT is also required for the repair of membrane lesions. Here, both cryo-EM and PI-staining give a strong impression of the presence of such lesions at the plasma membrane of cells lacking the ESCRT component Bro1, probably explaining in part the sick phenotype of the *bro1Δ* mutant. As such, our data strongly suggest that the role of ESCRT for plasma membrane repair, which so far was only demonstrated in mammalian cells, is evolutionary well-conserved. Interestingly, we found that the disruption of the plasma membrane integrity in the *bro1Δ* strain is dramatically exacerbated upon expression of αAβ_42_ and that this came along with the deterioration of the ER and an almost lethal phenotype. This is intriguing for several reasons. It demonstrates that when the fully processed Aβ_42_-linker-GFP arrives at the plasma membrane, the peptide further aggravates plasma membrane disruption, which given the observed effect on the ER, might well be involving fusion of secretory vesicles that contain disordered membranes. The fact that our screens retrieved the KO strain lacking *SSO2* as suppressor of αAβ_42_ toxicity favors the last possibility. Indeed, *SSO2* encodes a plasma membrane t-SNARE that is required for fusion secretory vesicles (Grote et al., 2000). Moreover, the data recapitulate observations made for AD where, as mentioned, Aβ_42_ was shown to introduce membrane lesions via different non-excluding mechanisms, including membrane lipid interaction, alterations in membrane fluidity, pore formation, or lipid oxidation (Eckert et al., 2000; Muller et al., 2001; Ambroggio et al., 2005; Rauk, 2008; Axelsen et al., 2011; Viola and Klein, 2015).

Given the effect of Aβ_42_ on plasma membrane integrity, the presence of this peptide also impacts on the overall cellular lipid homeostasis. In fact, extensive lipid alterations are implicated in the AD disease pathology but it is still a matter of debate whether such alterations are the cause or the consequence of AD (Grosgen et al., 2010; Xiang et al., 2015; El Gaamouch et al., 2016). In yeast, the expression of Aβ_42_ has been linked to a transcriptional upshift of key regulators of lipid metabolism as well as an enhanced formation of lipid droplets (Chen et al., 2017). Our screens with the yeast deletion collection and our Nile Red stainings support the link between Aβ_42_ and lipid metabolism. Intriguingly, a recent study demonstrated lipid droplet formation to be an adaptive response to an acute lipid imbalance in yeast cells. The same study then also showed that the biogenesis of these droplets occurs at ER aggregates (Vevea et al., 2015), which we believe to correspond to the ER-associated foci and filamentous structures seen when yeast cells express toxic forms of αAβ_42_, as mentioned. Notably, also in transgenic mouse models of AD an enhanced lipid droplet formation is observed (Hamilton et al., 2010; Yang et al., 2014), again underscoring the relevance of the data obtained in yeast.

## Author Contributions

GF, CM, HV, MV, and NT performed experiments and analyzed data. GF and JW wrote the draft. CDV, CC, and JW contributed conception and design of the studies. CC and JW corrected and edited the draft.

## Conflict of Interest Statement

The authors declare that the research was conducted in the absence of any commercial or financial relationships that could be construed as a potential conflict of interest.

## Footnotes

1 https://www.yeastgenome.org/cgi-bin/GO/goSlimMapper.pl
