# Supplementary material for: The Impact of ESCRT on Aβ1-42 Induced Membrane Lesions in a Yeast Model for Alzheimer’s Disease
Source: Front Mol Neurosci. 2018 Nov 5;11:406. doi: 10.3389/fnmol.2018.00406 (PMC6230623; doi:10.3389/fnmol.2018.00406)
Supplement: Supplementary file 1 [file Data_Sheet_1.PDF]

Supplementary Figure S1

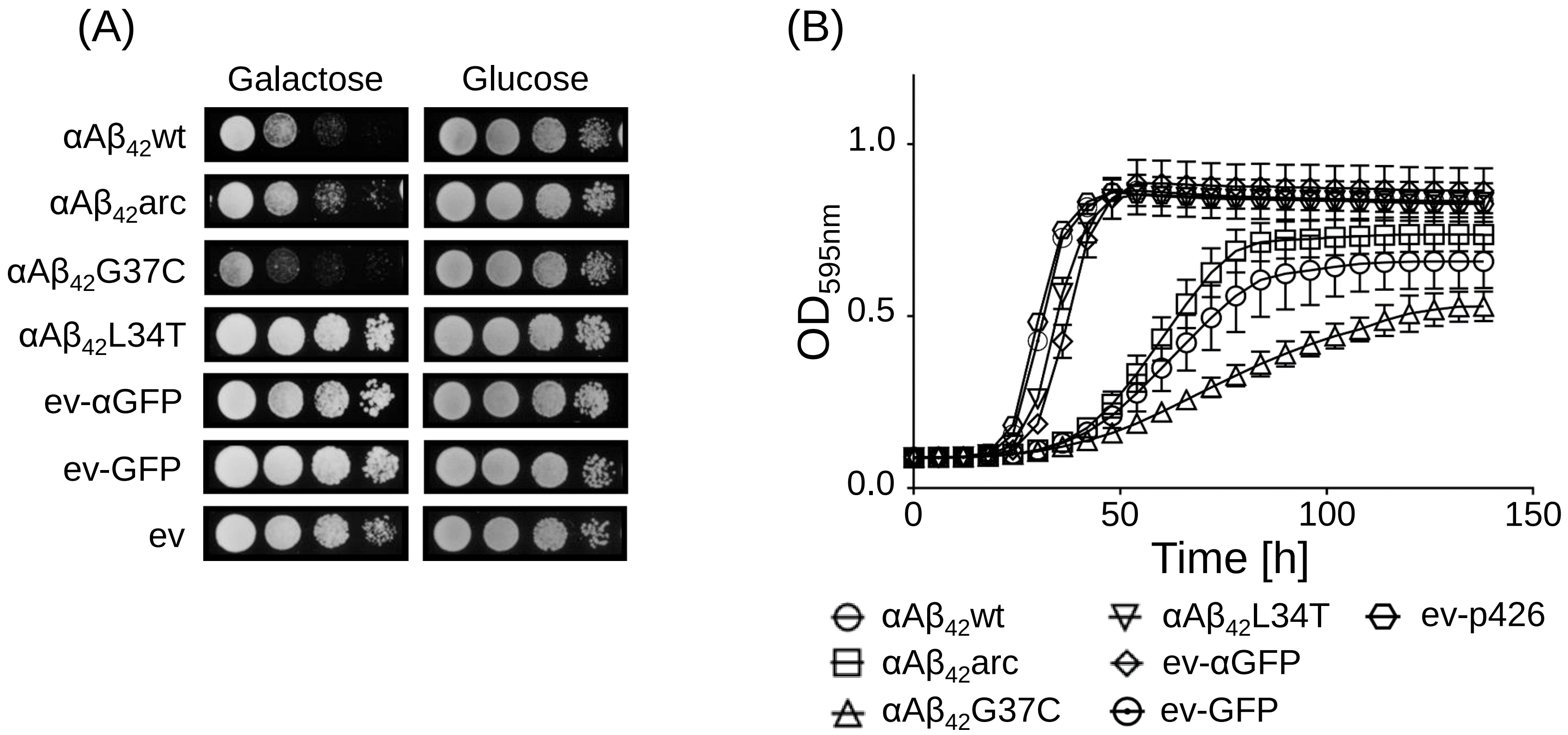

Supplementary Figure S1: Growth analysis of BY4742 allowing for expression of different αAβ_{42} mutants and controls.

BY4742 wild-type yeast strain transformed with plasmids carrying αAβ_{42}wt, αAβ_{42}arc, αAβ_{42}G37C, αAβ_{42}L34T and αGFP, GFP and an empty vector and (A) spotted on medium containing glucose to suppress and galactose to induce gene expression and (B) grown in liquid medium allowing for gene expression. Error bars represent standard deviations of at least four independent transformants.

Supplementary Figure S2

(A)

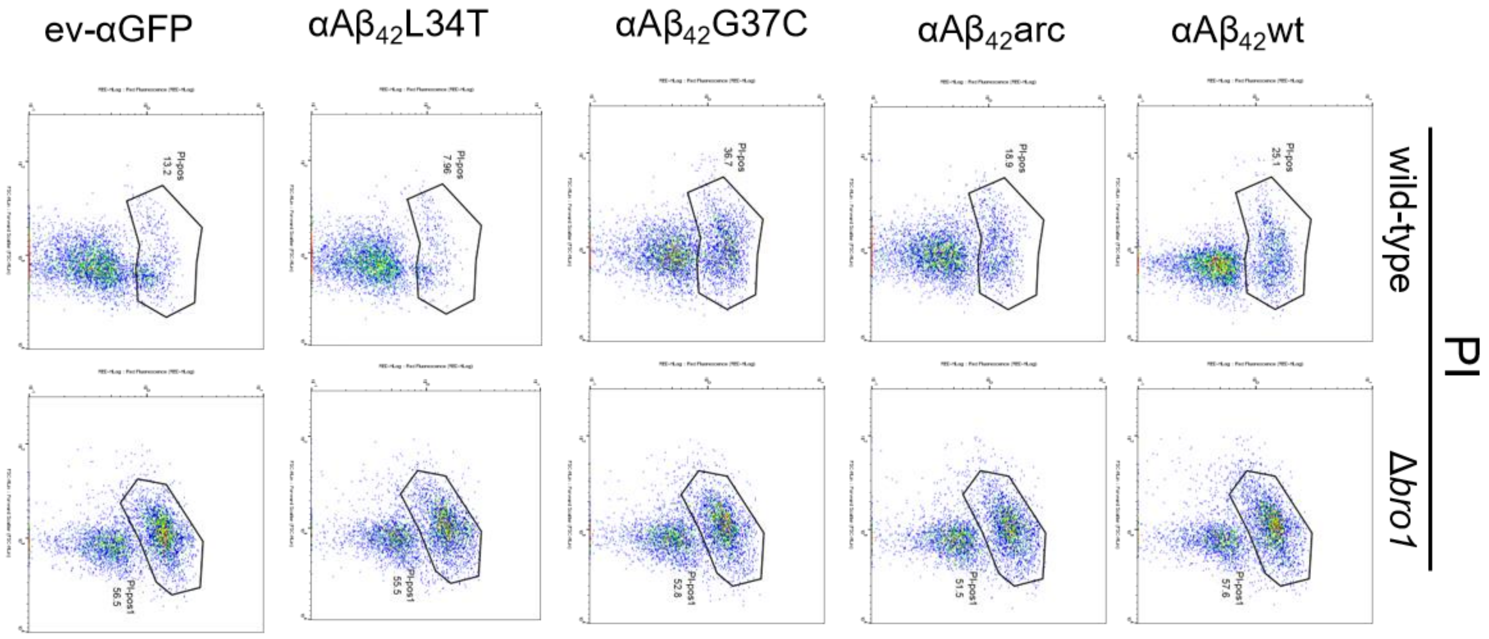

(B)

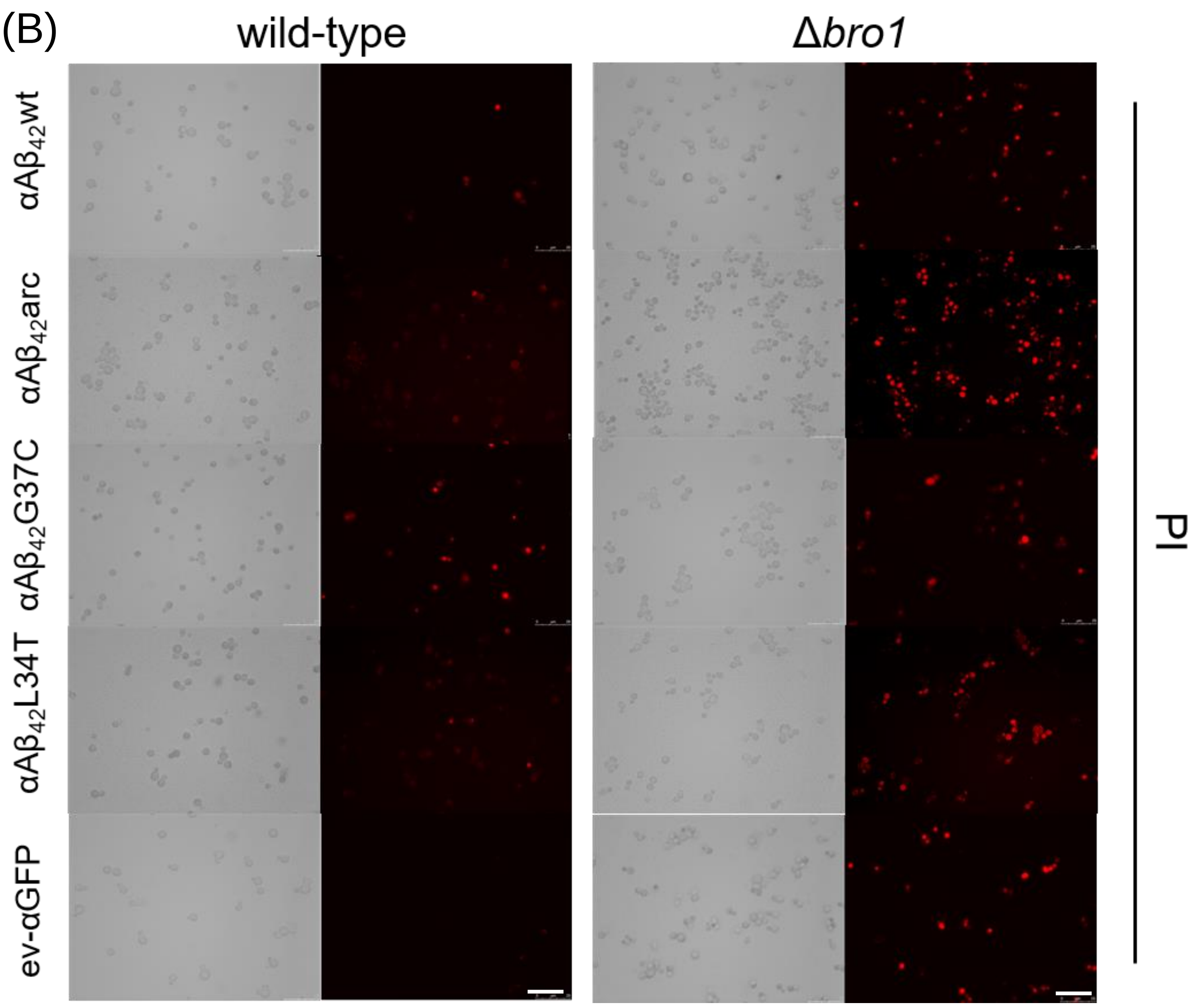

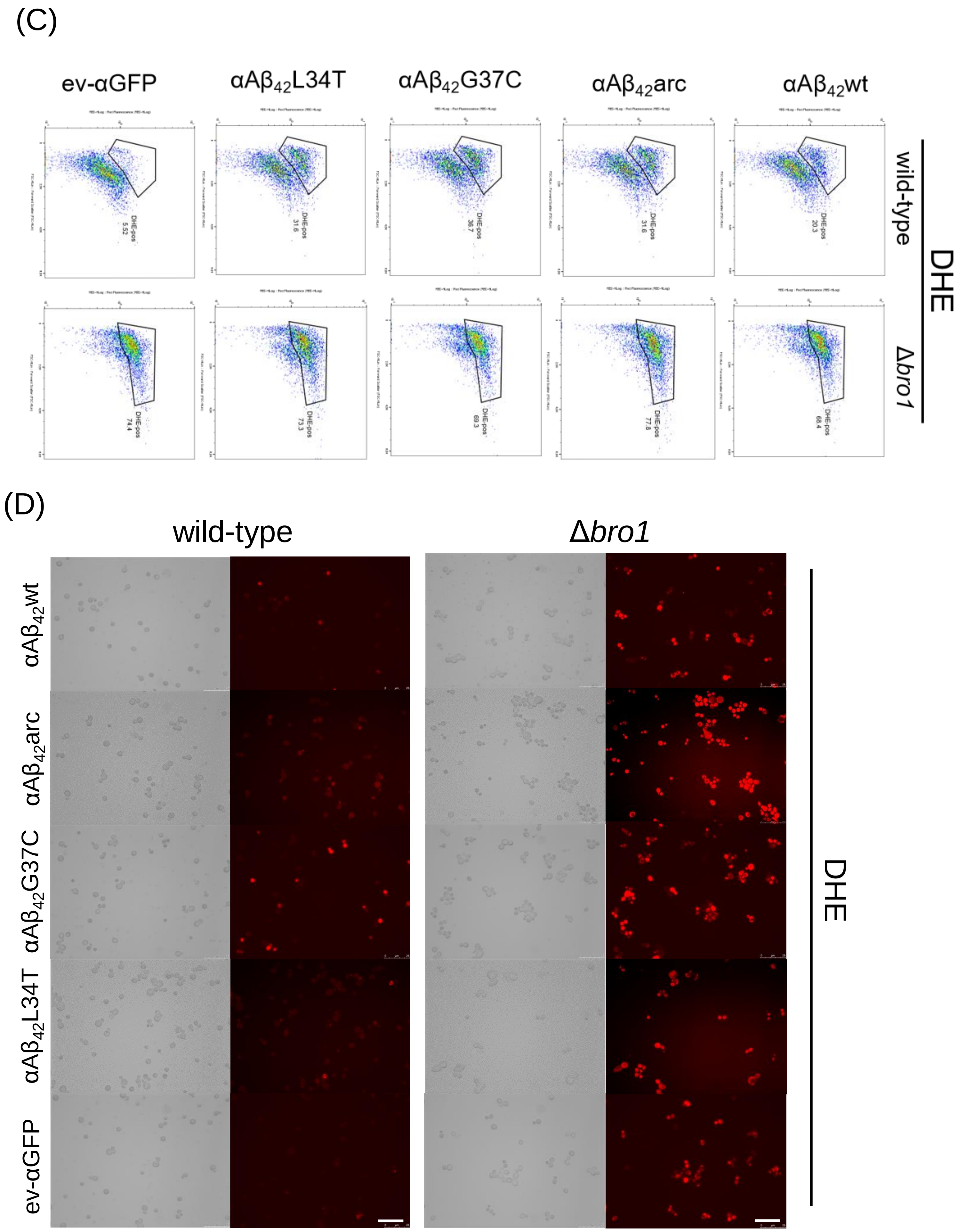

Supplementary Figure S2: Gating schemes and microscopy pictures of DHE and PI stained cells.

BY4742 wild-type and *bro1*Δ strains transformed with plasmids carrying $\alpha A\beta_{42}$wt, $\alpha A\beta_{42}$arc, $\alpha A\beta_{42}$G37C and ev-αGFP and stained with (A, B) propidium iodide (PI) and (C, D) dihydroethidium (DHE). Cytometry gating schemes for (A) PI and (C) DHE stained cells, microscopy pictures (left panels brightfield, right panels epifluorescence) of (B) PI and (D) DHE stained cells. Scale bars represent 25µm.

Supplementary Figure S3

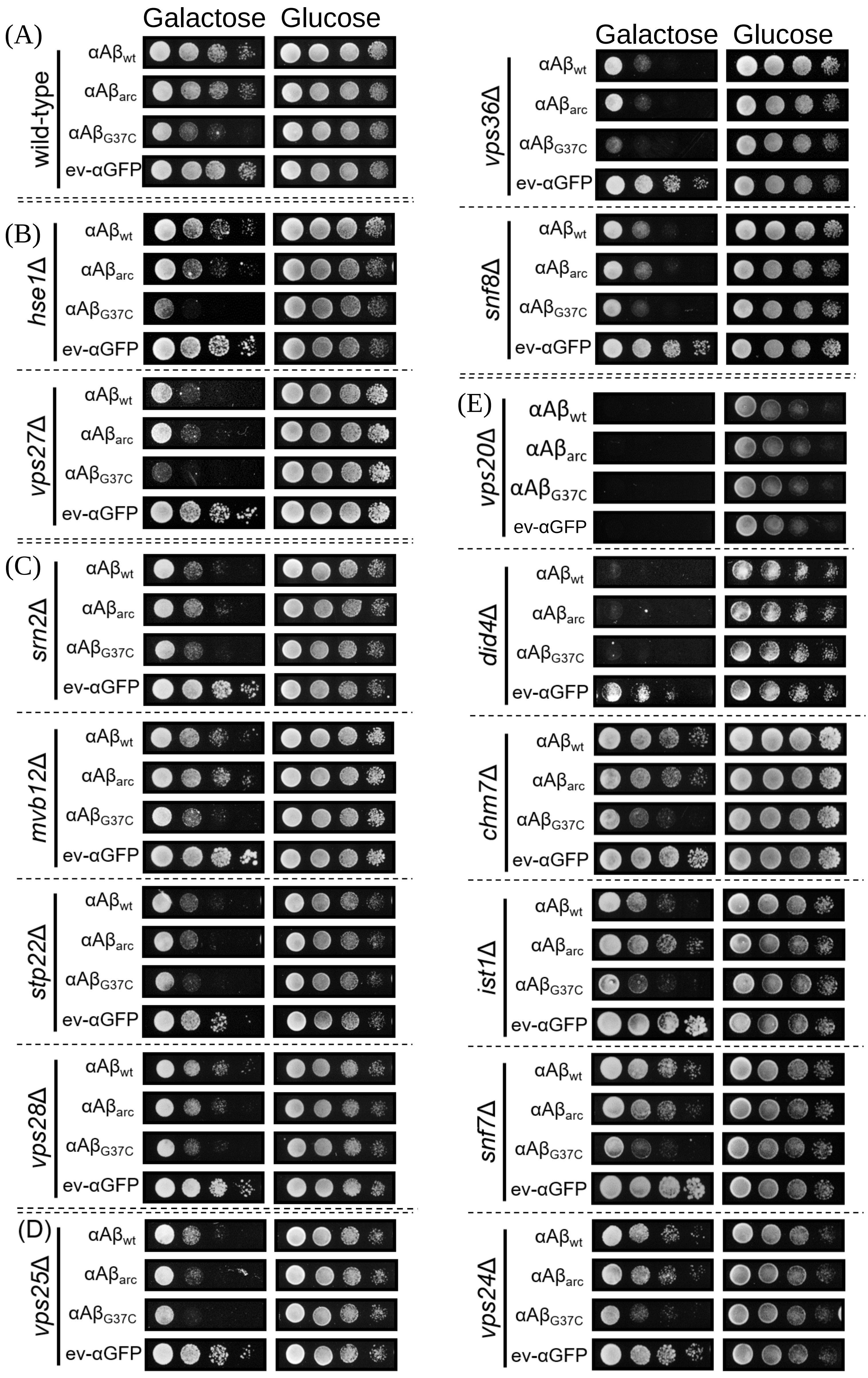

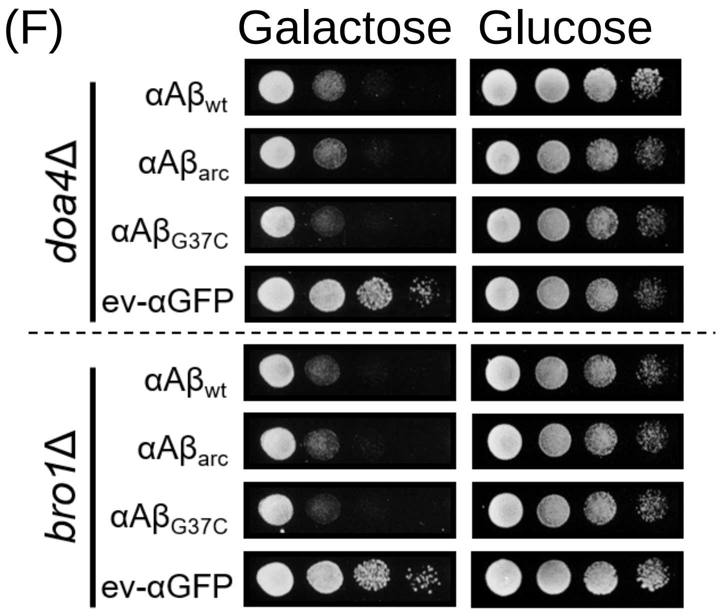

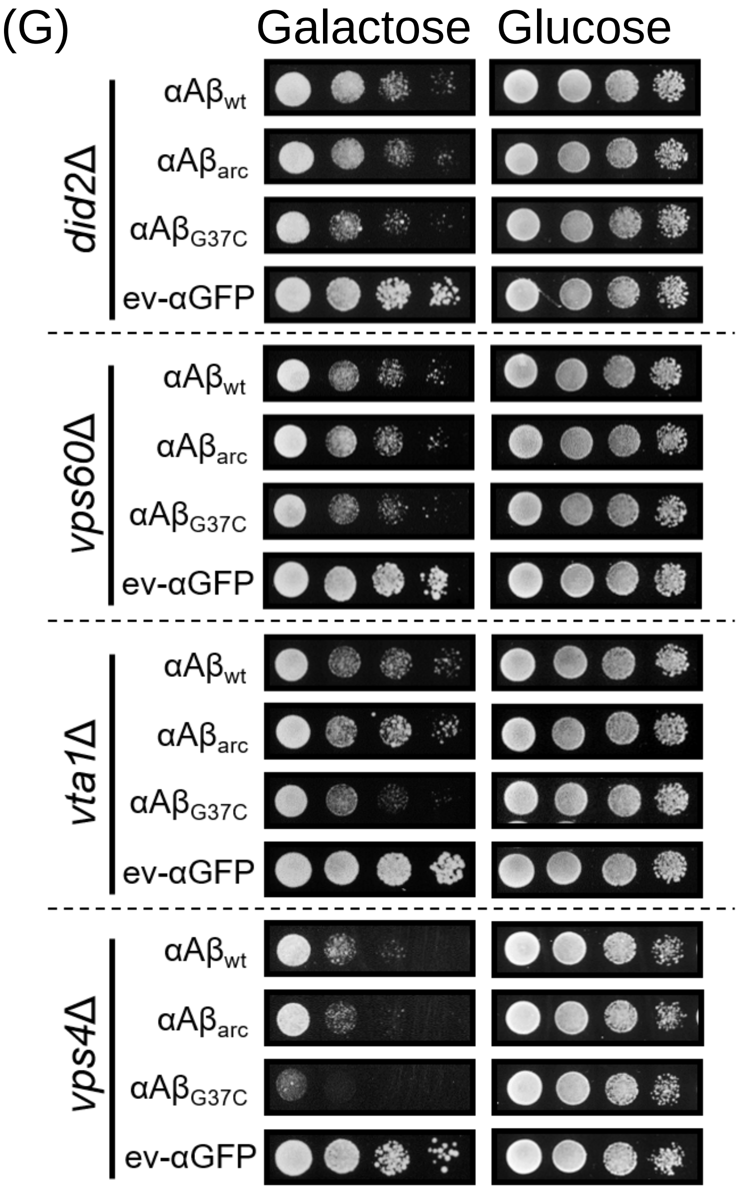

Supplementary Figure S3: Spot assays of strains knocked out for ESCRT key factors.
BY4742 wild-type strain and strains knocked-out for ESCRT key factors transformed with plasmids carrying $\alpha A\beta_{42}$wt, $\alpha A\beta_{42}$arc, $\alpha A\beta_{42}$G37C and ev-αGFP and spotted on medium containing glucose to suppress and galactose to induce gene expression. (A) BY4742 wild-type strain and knock-outs for factors of (B) ESCRT-0, (C) ESCRT-I, (D) ESCRT-II, (E) ESCRT-III, (F) ESCRT-III accessory and (G) ESCRT-III dissociation.

Supplementary Figure S4

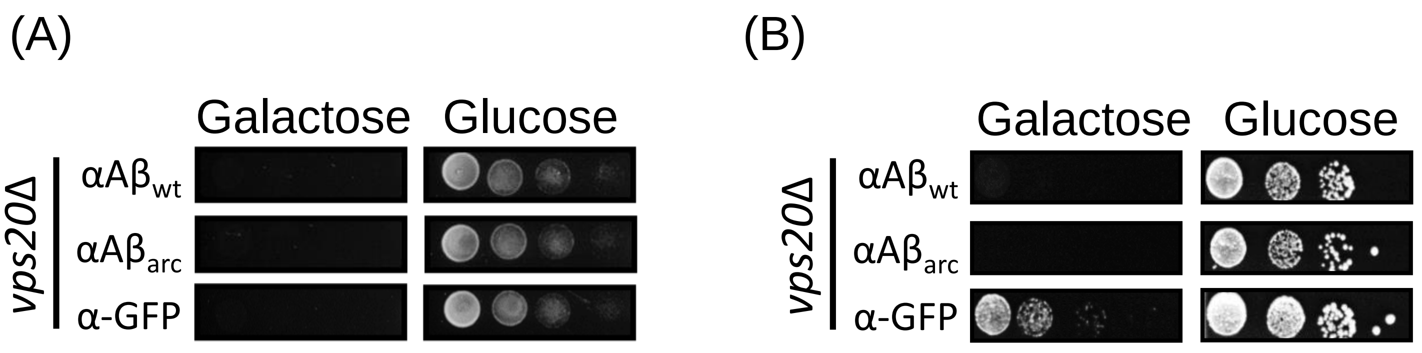

Supplementary Figure S4: A strain knocked out for *VPS20* does not grow on minimal medium supplemented with galactose.
BY4742 wild-type strain and strains knocked-out for ESCRT factor *VPS20* transformed with plasmids carrying $\alpha A\beta_{42}$wt, $\alpha A\beta_{42}$arc and ev-αGFP and spotted on medium containing glucose to suppress and galactose to induce gene expression. (A) Strains grown on minimal medium with 5,0 g/l of ammonium sulfate and supplemented with galactose containing, no growth can be observed. (B) Strains grown on galactose-containing minimal medium with increased concentrations of ammonium sulfate. Under these conditions also the control strain can grow.
